# Prevalence of Geriatric Syndromes in Elderly Cancer Patients Receiving Chemotherapy

**DOI:** 10.1155/2020/9347804

**Published:** 2020-02-22

**Authors:** Panita Limpawattana, Kusuma Phimson, Aumkhae Sookprasert, Wichien Sirithanaphol, Jarin Chindaprasirt

**Affiliations:** ^1^Division of Geriatric Medicine, Department of Medicine, Khon Kaen University, Khon Kaen 40002, Thailand; ^2^Department of Medicine, Khon Kaen University, Khon Kaen 40002, Thailand; ^3^Division of Medical Oncology, Department of Medicine, Khon Kaen University, Khon Kaen 40002, Thailand; ^4^Division of Urology, Department of Surgery, Faculty of Medicine, Khon Kaen University, Khon Kaen 40002, Thailand

## Abstract

The number of elderly patients with cancer is growing. Our study goals were to determine the prevalence of geriatric syndromes in elderly cancer patients receiving chemotherapy and its related factors using a basic geriatric screening tool. A cross-sectional study using the basic geriatric screening tool was conducted to survey geriatric problems in a population of elderly cancer patients receiving chemotherapy. There were 85 participants who were ≥60 years old. Descriptive statistics and regression analyses were used. The prevalence of having at least one geriatric syndrome was 58.8% (50 out of 85 cases). Depression was the most common component both in male and female patients. Age ≥65 years old was significantly associated with the geriatric syndrome (AOR 4.23, *p*=0.018), and a factor associated with depression was underweight (BMI<18.5 kg/m2) (AOR 13.2, *p*=0.003). In summary, geriatric syndromes are common in elderly cancer patients. Screening for geriatric syndrome adds substantial data on the assessment of elderly cancer patients, even those with a good performance status.

## 1. Introduction

As the number of older individuals is increasing in Thai society, it is expected that the proportion of elders would be 30 percent by the year 2030, and it is the fastest growing segment of the entire population [1].

The incidence of elderly cancer is also increasing since aging is one of the important risk factors for cancer. Between 1993 and 2012, the proportion of elderly cancer increased from 30 percent to 46 percent from the first to second decade [2].

There are many aspects to consisder when treating elderly cancer patients: not only the limitation in organ functions but also physicians have to deal with altered drug metabolism, polypharmacy, frailty, and comorbidity [3]. Using just general health condition evaluation such as Eastern Cooperative Oncology Group (ECOG) performance status to assess the older adults is not enough [4].

Geriatric syndromes are clinical conditions that are commonly found in older adults, especially in those who are frail [3–5]. These include but not limited to visual and hearing problem, urinary incontinence, falls, depression, dementia, delirium, and osteoarthritis of the knee. The conditions are common even in the community-dwelling older adults and are associated with increased risk for mortality [6]. In cancer patients, geriatric syndromes can complicate cancer treatment and affect the quality of life, overall mortality, and increasing the burden of caregivers [7].

Assessment of geriatric syndromes was found to be a crucial thing when treating the elderly with cancer; however, it is time consuming, and it is not feasible to do the comprehensive geriatric assessment in a busy oncology clinic. Screening for geriatric syndrome would be a good option to help clinicians plan cancer treatment for the elderly in various clinical settings [8, 9]. There are limited studies regarding geriatric syndromes using screening assessment particularly in cancer patients in Thailand. Therefore, the goals of this study were to determine the prevalence of geriatric syndromes in elderly cancer patients and its related factors using this scale.

## 2. Materials and Methods

This was a cross-sectional study that included cancer patients who were 60 years old or over who visited the chemotherapy clinic at Srinagarind Hospital, Khon Kaen University, Thailand, from November 2018 to January 2019.

### 2.1. Definition of Geriatric Syndromes

The term “geriatric syndrome” is used to define multiple conditions in elderly that do not fit into any specific disease; for example, dementia, delirium, falls, frailty, and urinary incontinence. The definition of geriatric syndromes assessed in this study comprised only six conditions: cognitive impairment, depression, osteoarthritis, falls, urinary incontinence, and dependent on functional daily activity.

### 2.2. Instrument and Procedure

Baseline patient data were collected after obtaining written informed consent. The demographic information consisted of age, sex, primary cancer site, and underlying diseases by medical record review and asking the patients. Body weight and height were recorded and were used to calculate the body mass index (BMI).

Basic geriatric screening [10], which was developed by the Ministry of Public Health of Thailand, consisted of 6 items and comprised six competencies: (1) memory (10-item recall test); (2) depression (2 items, “Did you often feel sad or depressed in the past 2 weeks?” and “Did you feel little interest or pleasure in the last 2 weeks?”); (3) osteoarthritis of knee (“Do you have knee pain?”); (4) falling (time up and go test); (5) urinary incontinence (“Have you lost urine and gotten wet that disturbed your daily life?”); (6) functional daily capacity (10 items, eating, grooming, toileting, bathing, dressing, bowel and bladder continence, transferring (in-out of bed/chair), getting around the house, and getting up 1 flight of stairs). The original version includes evaluation of oral health and vision, but they were excluded from our study. If the screening result was positive, the patient was referred to a physician for further examination.

### 2.3. Sample Size Calculation

Sample size calculation was based on the primary objective of this study, which was to ascertain the estimated prevalence of the geriatric syndrome in cancer patients. The estimated prevalence of 22–60% was derived from previous similar studies [5, 11]. A formula for estimating a population proportion with specified absolute precision was used to calculate this. It was determined that a sample size of at least 80 participants would be sufficient to achieve the required significance level of 0.05.

### 2.4. Statistical Analysis

Baseline data were presented as percentage, mean, and standard deviation (median and interquartile range if the distribution of the data was not normal). Univariable and multivariable regression analyses were utilized to determine the effects of factors associated with at least one geriatric syndrome. In univariable analysis, crude odds ratios (ORs) and 95% confidence intervals (CIs) were used to consider the strength of association. Factors with a *p* value of <0.20 or were found to be related in the literature review were then entered into a multiple logistic regression model. A *p* value of <0.05 was considered to indicate statistically significant differences, and adjusted ORs (AORs) and 95% CIs were used to determine the strength of association. All data analyses were carried out using STATA version 10.0 (StataCorp, College Station, TX, USA).

Ethical approval was provided by the Khon Kaen University Faculty of Medicine Ethics Committee as instituted by the Declaration of Helsinki.

## 3. Results

### 3.1. Prevalence of Geriatric Syndrome in Cancer Patients

**Figure 1:**
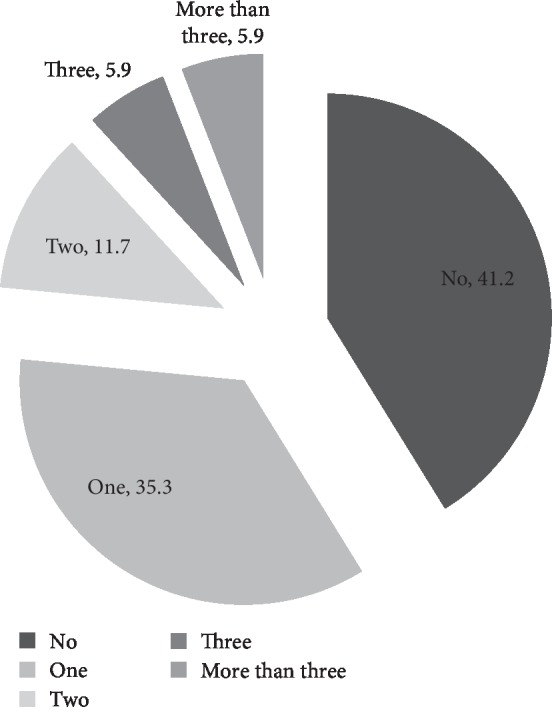
The number of geriatric syndrome in elderly patients (≥60 years old).

A total of 85 participants were enrolled. The prevalence of having at least one geriatric syndrome in patients aged ≥60, ≥65, and ≥70 years were 58.8%, 65.2%, and 69.0%, respectively. Twenty patients (23.5%) were found to have two or more geriatric syndromes as shown in Figure 1.

### 3.2. Distribution of Geriatric Syndromes Components by a Screening Method

**Figure 2:**
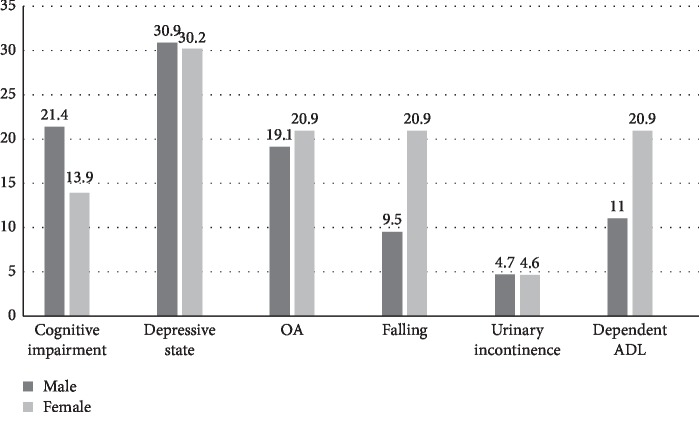
Geriatric syndrome in elderly patients.

The depressive condition, the most common geriatric syndrome in both male and female elderly patients, accounted for 30.6% of the overall cohort. Female patients exhibited more falling (OR 2.51, *p*=0.15) and dependent in ADL (OR 1.96, *p*=0.27) than male patients (Figure 2).

### 3.3. Factors Associated with Geriatric Syndromes

Baseline characteristics are listed in Table 1. Patients with the geriatric syndrome (GS) were slightly older than those without GS (68 vs. 66 years old). The proportion of underweight (BMI <18.5 kg/m2) was higher in GS (18% vs. 11.4%). Although most of the patients in this cohort suffered from gastrointestinal/hepatobiliary cancer, the BMI between patients with GI and non-GI cancer was similar (20.9 vs. 21.6 kg/m2). Diabetes was more common in GS patients (26% vs. 14.3%). Following the univariable analysis, the age of ≥65 years, diabetes, and GI cancer were entered into the multiple regression models. Only the age of 65 or older was found to have a statistically significant association with having at least one geriatric syndrome (Table 2).

### 3.4. Factors Associated with Depression

Depression was found to be a common problem in elderly cancer patients. After adjusted for BMI, type of cancer, and diabetes in the multivariate regression models, only underweight was a significant factor associated with depression as shown in Table 3.

## 4. Discussion

In this cohort, older adults with a cancer diagnosis had a high prevalence (58.8%) of having at least one geriatric syndrome by the basic geriatric screening tool. After adjustment for clinical factors, age is a statistically significant associated with the geriatric syndrome. Aging is associated with progressive decline in multiple organs and frailty [3, 4, 12].

The prevalence was comparable to those reported from community-dwelling older adults with a cancer diagnosis in the USA of 60.3% [5]. When considering only those older than 70 years old, the number was 69.0% which was similar to the study from Belgium and Netherland [13, 14].

The prevalence of specific geriatric syndromes ranged from 4.6% for urinary incontinence to 30% for depression. When compared to those with noncancer elderly in a study from Taiwan [8], the rate of depression was higher (30% vs 13%) in our cohort, and urinary incontinence was lower (4.6% vs 30%). A cancer diagnosis is the strongest factor associated with depression [5], so it is not surprising that those with cancer would have a higher risk of depression. For the urinary incontinence, in our cohort, we included only good performance status patients who were fit enough to get chemotherapy. Therefore, the rate of incontinence is lower than the general population.

It was found that females were more likely to develop depression than male patients [12]; however, this difference was not depicted in our study. Underweight patients (BMI <18.5 kg/m2) were significantly associated with the depressive state when compared to normal or overweight patients. This is particularly true and could be both the cause and result of depression. Depression decreases appetites and reduces food intake, vice versa, patients with cancer cachexia could affect overall health status and mood resulting in depression [15, 16]. Therefore, screening for nutritional status is also critical in treating cancer especially in older adults [17].

Several screening tools have been proposed to detect depression, functional disabilities, and dementia in a primary care setting [8, 9]. In cancer, however, there are limited reports. Basic geriatric screening is brief, easy-to-use, and simple, and it could be done by a nonphysician staff. However, one should keep in mind that a negative screening could not completely rule out the presence of depression. There was a report of novel interventions to deliver geriatric assessment through a mobile application, but the sample size was small [18].

There were limitations that should be considered. First, patients who were enrolled in this cohort were those who had a good performance status of at least 0 or 1 in order to be able to receive chemotherapy. This might not reflect the actual population of elderly cancer, and the number of geriatric syndromes would be underestimated. Second, this cancer sample was heterogeneous, including both hematologic and solid tumours. Third, the factors affecting the depressive state and functional status such as staging of neoplasm, chemotherapy regimen, and toxicity were not taken into account. Despite the limitations, this study demonstrated the high prevalence of geriatric syndromes in elders with cancer even with good performance status. These conditions are challenges for clinicians treating this population.

## 5. Conclusions

The geriatric syndrome is common in elderly cancer patients, and it is a challenge for oncologists. Incorporating easy-to-use screening tool to detect geriatric syndrome in elderly cancer patients is feasible and could have significant benefits in the real-life clinic.

## Figures and Tables

**Table 1:** Baseline characteristics and comparison of subjects with and without geriatric syndrome using univariable analysis.

	GS (*n* = 50)	Without GS (*n* = 35)	Unadjusted OR	95% CI	*p* value
Age < 65	5 (31.3)	11 (68.7)	4.12	1.28–13.26	0.017^*∗*^
Age ≥ 65	45 (65.2)	24 (34.8)			

Male, *n*(%)	25 (50.0)	18 (51.4)	0.94	0.40–2.24	0.90

Weight (kg), mean (SD)	52.3 (8.6)	53.2 (8.6)	0.99	0.94–1.04	0.61

BMI (kg/m^2^)					
<18.5	9 (18)	4 (11.4)	1	—	—
18.5–22.9	28 (56)	22 (62.9)	0.57	0.15–2.08	0.39
≥23.0	13 (26)	9 (25.7)	0.64	0.15–2.74	0.55

Primary cancer					
(1) Lung	3 (6)	3 (8.6)	1	—	—
(2) GI/hepatobiliary	25 (50)	22 (62.9)	1.14	0.21–6.22	0.88
(3) Leukemia	0	2 (5.7)	1	—	—
(4) Breast cancer	1 (2)	1 (2.9)	1	—	—
(5) Other	21 (42)	7 (20.0)	2.99	0.20–4.95	0.23

DM, *n*(%)	13 (26)	5 (14.3)	2.11	0.68–6.58	0.20

HT, *n*(%)	14 (28)	9 (25.7)	1.12	0.42–2.99	0.81

DLD, *n*(%)	8 (16)	4 (11.4)	1.48	0.85–2.15	0.55

GS, geriatric syndrome; OR, odds ratio; SD, standard deviation; BMI, body mass index; GI, gastrointestinal; DM, diabetes mellitus; HT, hypertension; DLD, dyslipidemia.

**Table 2:** Factors associated with geriatric syndrome using multivariable analysis.

Factors	Adjusted odds ratio (95% CI)	*p* value
Age ≥65	4.23 (1.28, 14.01)	0.018^*∗*^
DM	1.77 (0.54. 5.82)	0.344
Non-GI cancer	1.80 (0.71, 4.61)	0.217

DM, diabetes mellitus; GI, gastrointestinal.

**Table 3:** Factors associated with elderly patients having depression by univariable and multivariable analyses.

Factors	Unadjusted OR (95% CI)	*p* value	Adjusted OR (95% CI)	*p* value
Age	1.03 (0.93, 1.13)	0.57	—	

Gender			—	
Male	1	—		
Female	0.97 (0.38, 2.43)	0.94	—	

BMI (kg/m^2^)				
Overweight (≥23)	1	—	—	—
Normal (18.5–22.9)	1.75 (0.50, 6.09)	0.38	2.32 (0.62, 8.69)	0.21
Underweight (<18.5)	7.2 (1.52, 34.14)	0.013	13.2 (2.37, 73.66)	0.003^*∗*^

Type of cancer			
GI	1	—	1	—
Non-GI cancer	2.13 (0.84, 5.45)	0.11	2.49 (0.88, 7.03)	0.08

DM	2.18 (0.74, 6.38)	0.16	3.13 (0.93, 10.55)	0.06

HT	0.74 (0.25, 2.17)	0.58		

DLD	1.16 (0.32, 4.25)	0.82		

## Data Availability

The data used to support the findings of this study are available from the corresponding author upon request.
